# Case Report: Early Neonatal EEG in Two Infants with Pallister Killian Syndrome (PKS)

**DOI:** 10.12688/hrbopenres.13493.1

**Published:** 2022-02-18

**Authors:** Carol M Stephens, Andreea M Pavel, Sean R Mathieson, Niamh McSweeney, Brian McNamara, Michael Moore, Geraldine B Boylan

**Affiliations:** 1 INFANT Research Centre, University College Cork, Cork, Ireland; 2 Department of Paediatrics and Child Health, University College Cork, Cork, Ireland; 3 Department of Neonatology, Cork University Maternity Hospital, Cork, Ireland; 4 Department of Paediatric Neurology, Cork University Hospital, Cork, Ireland; 5 Department of Neurophysiology, Cork University Hospital, Cork, Ireland; 6 Department of Radiology, Cork University Hospital, Cork, Ireland

**Keywords:** Neonatal seizures, electroencephalography (EEG), Pallister-Killian Syndrome, epilepsy

## Abstract

Pallister Killian Syndrome (PKS) is a rare genetic disorder caused by a mosaic tetrasomy of the short arm of chromosome 12. The syndrome is characterised by typical craniofacial dysmorphism, congenital anomalies and intellectual disability. Epilepsy is a known complication, with onset usually occurring in early childhood and characterised most commonly by spasms and myoclonic seizures. To the best of our knowledge, there have been no cases describing the early neonatal EEG in PKS and  electrographic seizures, to date. Here, we report two cases of PKS presenting in the neonatal period with distinctive EEG features and seizures.

## Introduction

PKS is a rare genetic disorder with an incidence of one per twenty-thousand births ^1, 2^. It is characterised by a distinct phenotype and can be associated with several complications. Epilepsy is a common neurological sequela, usually presenting in childhood. Few cases of neonatal onset seizures have been described to date and no detailed electrographic descriptions are available.

 Here, we present two cases with similar phenotypical characteristics along with early EEG findings. Identification of distinctive electrographic features can aid in recognition and diagnosis of Pallister Killian Syndrome. This can ultimately lead to earlier targeted interventions. Genetic counselling can be provided, where appropriate, along with earlier engagement with multi-disciplinary teams.

## Case presentations

### Case one

**Figure 1.**
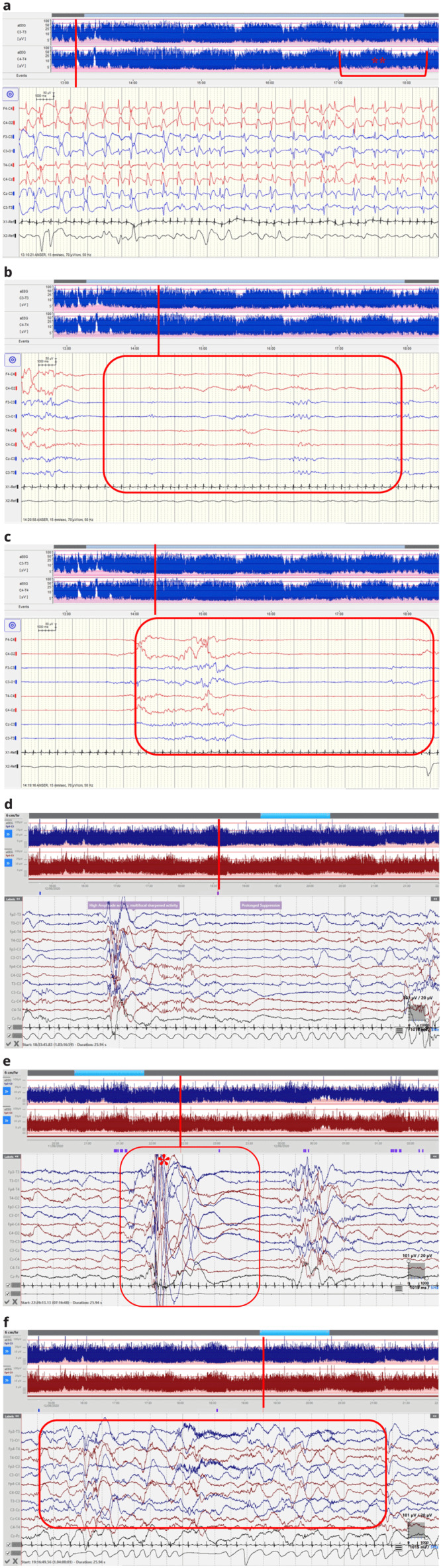
( **a**– **f**) EEG recordings. *(EEG displayed at sensitivity 70μV/cm, time-base 15mm/sec. aEEG displayed at 6cm/sec)*. ( **a**) Case One: Electrographic seizure at 59 hrs of life consisting of generalised sharp and slow wave complexes with clear aEEG correlate (*). Sleep-wake cycling poorly defined at the beginning of the recording but improves over time as demonstrated on the aEEG (**). ( **b**) Case One: Dysynchrony in Quiet Sleep at 61hrs of life. ( **c**) Case One: Prolonged discontinuity in Quiet Sleep at 61 hrs of life. ( **d**) Case Two: cEEG at 56 hrs of life showing prolonged suppression in Quiet Sleep. High amplitude activity with multifocal sharp waves. ( **e**) Case Two: Myoclonic jerk (during Quiet sleep at 35 hours of life showing high amplitude movement activity followed by brief suppression. ( **f**) Case Two: Active Sleep showing continuous activity but excessively low frequencies on the background pattern for gestational age at 37 hours of life (example highlighted).

Case 1 was a term female infant born via emergency lower segment caesarean section (LSCS) for failure to progress at forty one weeks and five days gestation. She was the first child of non-consanguineous Caucasian parents. An antenatal MRI identified mild left ventriculomegaly and shortened long bones. At birth, she had dysmorphic features; microphthalmia, a high arched palate, low set ears, micrognathia and roving eye movements. She had a good suck, but no rooting or grasp, she had an asymmetrical Moro reflex and increased upper limb tone with fisting. Reflexes were present but she had marked central hypotonia. She was admitted to the neonatal unit with hypoglycaemia (1.1mmol/L) and central hypotonia. At fifty-seven hours of life, she had generalised clonic seizures during which blood sugar was normal. Continuous video EEG (cEEG) monitoring (Nihon Kohden) was commenced using scalp electrodes at positions F3,F4,C3, C4,T3,T4,O1,O2,CZ at 58 hours. ECG and respiratory movements were also recorded simultaneously. At commencement of EEG recording, three electrographic-only seizures were captured that had an abrupt onset and termination ( Figure 1a). Whilst, the first seizure was brief in duration (44 seconds), the second two each lasted over two minutes. Seizures consisted of sharp and slow wave complexes with onset in the left or right central regions with secondary generalisation. Sleep wake cycling was present but poorly defined over the initial recording but became better defined as time progressed. Quiet sleep (QS) phases were characterised by dysynchrony with long periods of suppression ( Figures 1b and 1c). A loading dose of 20mg/kg of Phenobarbitone was administered and the infant remained seizure free in the neonatal period. Follow up EEG on day eleven showed an encephalopathic background with prolonged periods of suppression, absence of sleep-wake cycles and ongoing dysynchrony.

**Figure 2.**
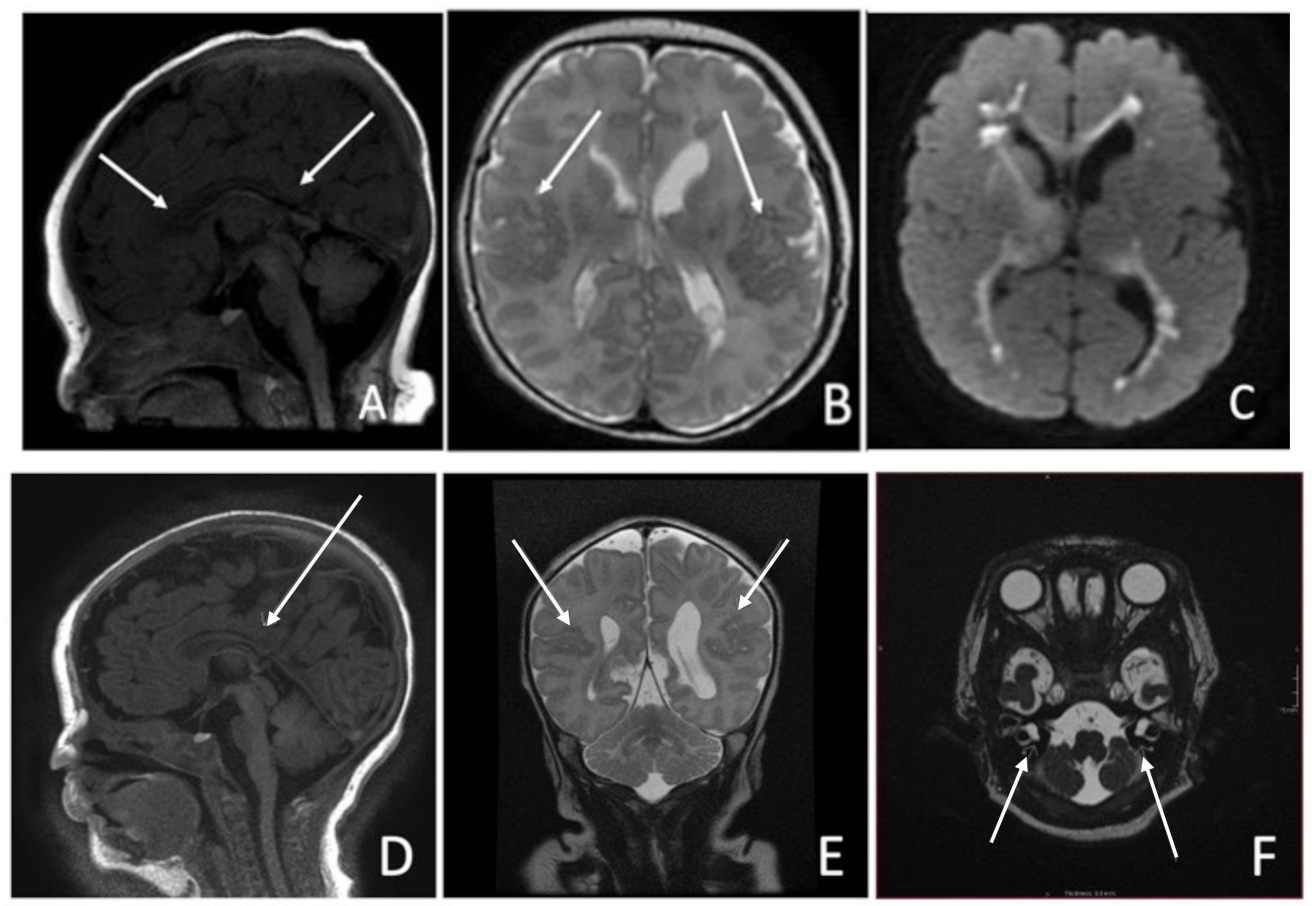
( **a**– **f**)- Brain MRI. Case One: ( **a**) Thinning of the body of the corpus callosum. The rostrum and splenium appear blunted, likely representing partial dysgenesis, ( **b**) Bilateral polymicrogyria in the posterosuperior aspects of the sylvian fissures, ( **c**) Marked restriction within the periventricular regions bilaterally - involving the heterotopic grey matter. Case Two: ( **d**) Sagital T1 View: Partial agenesis of Corpus Callosum posteriorl, ( **e**) Coronal T2 View: Mild bilateral perisylvian microgyria, ( **f**) Axial T2 View: Bilateral Cystic dilatation of vestibules.

Cranial ultrasound on day one was unremarkable. MRI on day three showed thinning of the corpus callosum with blunting of the rostrum and splenium ( Figure 2a), bilateral perisylvian polymicrogyria ( Figure 2b) and significant diffusion restriction in the periventricular regions bilaterally ( Figure 2c). Echocardiogram on day seven revealed an Atrial Septal Defect(ASD) Secundum. She failed otoacoustic and auditory brainstem new-born hearing screen on day twenty-nine and subsequently required bilateral hearing aids. Given the constellation of features described, a genetic disorder was considered the most likely diagnosis. Microarray identified a gain in the short arm of chromosome 12 at 12p13.33 to p11.1, consistent with PKS.

At twelve months, she developed myoclonic seizures characterised by single jerks of the upper and lower limbs symmetrically accompanied with eye rolling or deviation to the left. EEG at the time showed spike and high amplitude slow wave discharge with a slower background pattern than expected. She was commenced on Levetiracetam but despite high dose treatment, seizures continued so she was changed to a combination of Sodium Valproate and Clobazam. Clobazam was discontinued due to over sedation. Current treatment at twenty one months is Sodium Valproate monotherapy and she is experiencing ten to twenty brief myoclonic jerks daily. She has global developmental delay. 

### Case two

The second case was a male infant, born via emergency LSCS for cord prolapse at thirty-eight weeks gestation. He was the third child born to non-consanguineous Caucasian parents. Both parents were carriers of the Connexin 26 gene and two elder daughters had hearing impairments, one of whom had a cochlear implant. Antenatally, there were initial concerns regarding ventriculomegaly but this had resolved on subsequent ultrasound scans. Following delivery, the infant was admitted to the neonatal unit for hypotonia and respiratory support via CPAP for suspected Transient Tachypnoea of the Newborn (TTN). He too, was treated for hypoglycaemia (0.8 mmol/L) which later resolved. Initial examination revealed dysmorphic features; a large open fontanelle, coarse, flat facies, ante-verted nares, a nuchal fold, shawl bifid scrotum with an abnormal raphe and a sacral dimple with a tuft of hair and asymmetrical skin folds. Neurologically, he had a weak palmar grasp, a reduced Moro reflex, a reduced suck with no rooting and was both, centrally and peripherally hypotonic with reduced reflexes.

At approximately twenty nine hours of age, tonic seizures were suspected characterised by episodes of desaturation and upper and lower left limb extension and stiffening. cEEG monitoring (Lifelines iEEG, Lifelines Neuro, UK) was commenced using scalp electrodes at positions FP3,FP4,C3, C4,T3,T4,O1,O2,CZ,PZ . ECG and respiratory movements were also recorded simultaneously. The background EEG was generally continuous initially with some evidence of sleep cycling. In QS the EEG was excessively discontinuous with bursts of high amplitude delta activity at 100–400 µV intermixed with sharp waves, alternating with periods of lower amplitude theta activity of less than 25µV lasting five-ten seconds ( Figure 1d). Frequent myoclonic jerks were associated with bursts of high amplitude movement activity on EEG but no clear seizures in quiet sleep ( Figure 1e). In active sleep and waking, the EEG was continuous but with excess delta activity for gestational age ( Figure 1f). EEG was recorded in two prolonged periods for eight of the subsequent 12 days with little improvement of the background pattern. Clinically, episodes became more variable over time. Initial clinical episodes prior to EEG recording were characterised by left arm and leg extension and stiffening. Later, these episodes evolved to myoclonic jerks but with no clear electrographic seizures. Clinical events reduced on administration of an initial load of 20mg/kg of Phenobarbitone, followed by a maintenance dose of 5mg/kg/day and Levetiracetam 60mg/kg/day. Follow up EEG on day forty one (twenty seven days corrected gestational age) showed diffuse low-voltage, slow wave theta and delta activity with periods of relative low amplitude. This was symmetrical over both hemispheres but there was asynchrony, with occasional sharpened features over the right frontal region and the left temporal region. Both clinical and electrographic features were suggestive of an early epileptic encephalopathy. 

Cranial ultrasound on day one suggested thinning of the corpus callosum along with a lobulated appearance of the left ventricle posteriorly. MRI Brain on day five showed partial agenesis of corpus callosum posteriorly ( Figure 2d) along with mild perisylvian polymicrogyria bilaterally ( Figure 2e). Bilateral vestibular dilatation was noted in the internal auditory meatus ( Figure 2f), previously described in relation to Connexin gene mutations. Hearing screening on day forty three (twenty nine days corrected gestational age) revealed no clear response bilaterally on automated otoacoustic emission testing but clear responses bilaterally for automated auditory brainstem testing. The infant subsequently required bilateral hearing aids. Echocardiogram on day four revealed a small Patent Ductus Arteriosus and a small ASD Secundum. Similar, to case one, an underlying genetic diagnosis was considered most likely in this case. Microarray confirmed the diagnosis of PKS with a large 34.5 gain-2 copy gain on chromosome 12-isochromosome-4 copies of short arm of chromosome 12. He was also found to be heterozygote for the pathogenic GJB2 mutation c.35del p.(Gly12fs) on the background of his mother possessing a connexin 26 mutation. This genetic finding in isolation was not felt to be sufficient to account for his hearing impairment alone, although a second, undetected DFNB1 change of clinical significance could not be ruled out. This would lead to the assumption that his hearing impairment was a result primarily of his underlying diagnosis of PKS. Complete resolution of clinical events was achieved in the neonatal period by day fourteen.

## Consent

Written informed consent for publication of the clinical information was obtained from both children’s legal guardian.

## Discussion

In our cases, herein described, an early genetic diagnosis was confirmed in both. The distinctive EEG features and abnormal backgrounds guided clinicians to consider an underlying genetic disorder early in the management process. Appropriate physicians and multi-disciplinary teams were involved in the care of both infants from an early stage and allowed for future focused, patient-specific follow-up.

PKS is a rare genetic syndrome secondary to a mosaic tetrasomy of the short arm of chromosome 12 ^3^. It is characterised by facial dysmorphism, congenital anomalies, global developmental delay and epilepsy. Our cases both presented in the neonatal period with varying combinations of the classical phenotype along with congenital anomalies (partial agenesis of the corpus callosum, a Patent Ductus Arteriosus and bilateral hearing impairments) and early epileptic encephalopathy. The initial hypoglycaemic episodes seen in both cases have been previously described in neonates with PKS ^4^ along with distinctive neuroradiology features of corpus callosum dysgenesis, microcephaly and perislyvian polymicrogyria ^5^, cardiac defects ^6^ and hearing impairment ^7, 8^ These cases are the earliest descriptions of EEG features of PKS that have been reported to date.

The incidence of epilepsy in association with PKS varies from 42%–72% ^8– 10^. Neonatal seizures are rare and only two reports have been published in the literature. The first was that of a neonate who had ‘staring episodes’ and myoclonic jerks but no further information was provided ^10^. The second case was that of a premature infant born at thirty two weeks gestation, which was also associated with respiratory distress and hypoglycaemia; similar to our second case ^11^. No details were available regarding seizure semiology or EEG characteristics in this case, however seizures were well controlled up until recurrence at fifteen months of age. Candee *et al*. report in their cohort, seizure onset by two years of age in 56% and by six years in 96% along with an association between PKS and refractory seizures ^9^. In a recent case series of thirteen Italian children with PKS, infantile spams were most common, with the earliest onset of seizures occurring at seven months of age and only four cases having seizure onset within the first twelve months of life ^4^.

Seizure semiology in PKS can vary from generalised, focal, myoclonic or spasms ^4, 12– 16^. Myoclonic epilepsy is common in PKS, having been reported in a case series of three patients by Ricci ^16^, 15/27 patients in Candee’s cohort ^9^, 9/22 by Blythe ^8^ and only 2/13 by Giordano ^4^. The earliest report of myoclonic seizures describe onset at ten months ^4^. EEGs show slow and disorganised background activity and frequent bursts of diffuse or multi-focal sharp waves ^4^. Seizures can be triggered by low frequency photic stimulation. Ricci *et al*. describe myoclonic epilepsy with photosensitivity in three patients. Both spontaneous and photo-induced jerks at low frequencies (1–6Hz) were recorded with spike and slow wave complexes in all three patients. Our electrographic recordings (though recorded much earlier) appear similar to previous reports with low frequency and multifocal sharp waves. Sleep-cycling was evident in the earliest EEG recordings of our patients though the background was excessively discontinuous in quiet sleep and excessively slow in active sleep for gestational age ( Figure 1a and 1b). The best pharmacological success for seizure control has been achieved with Sodium Valproate or Levetiracetam or a combination of both ^4, 9, 16^. Our first case has trialled both to date, whilst the second is being managed successfully on Levetiracetam alone.

These cases highlight the importance of early consideration of an underlying genetic disorder in infants presenting with highly abnormal background EEG patterns in the neonatal period. Focused diagnostic methods and subsequent optimal targeted treatments may lead to improved management and future outcomes. Early directed treatment is key in the management of neonatal seizures in an effort to minimise high seizure burden and the administration of multiple anti-epileptic drugs, both of which are associated with poor outcomes ^17^. Furthermore, genetic counselling may be required, where appropriate, for affected families.

To conclude, PKS is a rare chromosomal disorder characterised by epilepsy, congenital anomalies, global development delay and facial dysmorphism. Here, we have described, the earliest EEG features associated with neonatal seizures in PKS. Epilepsy types vary, with spasms and myoclonic seizures most common in infancy. Early electrographic features of myoclonic seizures in infancy are characterised by an encephalopathic pattern with frequent bursts of diffuse or multi-focal sharp waves. Seizures can be refractory with most pharmacological success achieved with Sodium Valproate or Levetiracetam or a combination of both ^4, 9, 16^.

## Data availability

All data underlying the results are available as part of the article and no additional source data are required.
