# Supplementary material for: Case Report: Early Neonatal EEG in Two Infants with Pallister Killian Syndrome (PKS)
Source: HRB Open Res. 2022 Feb 18;5:14. [Version 1] doi: 10.12688/hrbopenres.13493.1 (PMC9530620; doi:10.12688/hrbopenres.13493.1)
Supplement: Peer review history

# Peer review: Reviewer response for version 1

**DOI:** 10.21956/hrbopenres.14716.r32904

**Published:** 2022-10-03

**Authors:** Fabrice Wallois

**Affiliations:** 1 Research Group on Multimodal Analysis of Brain Function, Jules Verne Picardie University, Amiens, France; 2 Inserm, Paris, France

Pallister Killian Syndrome (PKS) is a rare genetic disorder for which there are no publications relating to EEG analysis. In this paper, two cases of PKS are reported in the neonatal period with distinctive EEG features and seizures. While only a few cases of neonatal onset seizures have been described to date, no detailed electrographic descriptions are available.

 This article is very well written. It describes with precision the electrographic events that accompany the seizures in this syndrome whose incidence is 1/20000. I have only minor comments to improve the manuscript.

- The background activity in the QS could be described with as much precision in case 1 as in case 2. Active sleep is not described in case 1. Clinical appearance at onset and during seizures could perhaps be better specified. A video could be attached in SI if available. Especially for case 2 where the question of the nature of the event aspect can be discussed.
- I would have made two figures (1 and 2) each for each patient by grouping: -seizure onset- generalization and end of seizure for patient 1 and the aspect of slow waves intricate spikes + clinical manifestation for patient 2. Then on the same figures (1 and 2) the AS and QS patterns.
- I would have made a table regrouping the clinical-critical EEG aspect during QS and during AS-MRI-treatment-Clinical outcome.

These are only minor comments.

Are enough details provided of any physical examination and diagnostic tests, treatment given and outcomes?

Yes

Is the background of the cases’ history and progression described in sufficient detail?

Yes

Is sufficient discussion included of the importance of the findings and their relevance to future understanding of disease processes, diagnosis or treatment?

Yes

Is the conclusion balanced and justified on the basis of the findings?

Yes

Reviewer Expertise:

Neurodevelopment

I confirm that I have read this submission and believe that I have an appropriate level of expertise to confirm that it is of an acceptable scientific standard.

# Peer review: Reviewer response for version 1

**DOI:** 10.21956/hrbopenres.14716.r31621

**Published:** 2022-03-21

**Authors:** Janette Mailo

**Affiliations:** 1 Division of Pediatric Neurology, University of Alberta, Edmonton, AB, Canada

With great interest I read this manuscript describing early clinical, electrographic and imaging characteristics of Pallister-Killian syndrome.

 The cases are well described with sufficient details, including clinical exam findings, in particular pertinent positive and negative findings, as well as good details of EEG/aEEG and imaging, to support discussion and conclusions. Discussion is well balanced between authors' findings and the available literature data to formulate conclusions stated by the authors. 

 Overall, this article is scientifically sound and well written. The discussion and conclusion will be helpful to neonatologists, pediatric neurologists, pediatricians and others caring for neonates and infants.

 Authors' suggestions when to consider genetic diagnosis are excellent and will be helpful to judiciously use costly genetic testing.

 I recommend this article for indexing.

Are enough details provided of any physical examination and diagnostic tests, treatment given and outcomes?

Yes

Is the background of the cases’ history and progression described in sufficient detail?

Yes

Is sufficient discussion included of the importance of the findings and their relevance to future understanding of disease processes, diagnosis or treatment?

Yes

Is the conclusion balanced and justified on the basis of the findings?

Yes

Reviewer Expertise:

Neonatal Neurology, Neonatal Neurocritical Care, Neonatal EEG and aEEG

I confirm that I have read this submission and believe that I have an appropriate level of expertise to confirm that it is of an acceptable scientific standard.
